# Discordant High-Gradient Aortic Stenosis: A Systematic Review

**DOI:** 10.3390/jcdd12070255

**Published:** 2025-07-03

**Authors:** Nadera N. Bismee, Mohammed Tiseer Abbas, Hesham Sheashaa, Fatmaelzahraa E. Abdelfattah, Juan M. Farina, Kamal Awad, Isabel G. Scalia, Milagros Pereyra Pietri, Nima Baba Ali, Sogol Attaripour Esfahani, Omar H. Ibrahim, Steven J. Lester, Said Alsidawi, Chadi Ayoub, Reza Arsanjani

**Affiliations:** Department of Cardiovascular Medicine, Mayo Clinic, 5777 East Mayo Boulevard, Phoenix, AZ 85054, USA; nadera.bismee@gmail.com (N.N.B.); abbas.mohammedtiseer@mayo.edu (M.T.A.); sheashaa.hesham@mayo.edu (H.S.); abdelfattah.fatmaelzahraa@mayo.edu (F.E.A.); farina.juanmaria@mayo.edu (J.M.F.); awad.kamal@mayo.edu (K.A.); scalia.isabel@mayo.edu (I.G.S.); pereyra.milagros@mayo.edu (M.P.P.); babaali.nima@mayo.edu (N.B.A.); attaripouresfahani.sogol@mayo.edu (S.A.E.); ibrahim.omar3@mayo.edu (O.H.I.); lester.steven@mayo.edu (S.J.L.); alsidawi.said@mayo.edu (S.A.); ayoub.chadi@mayo.edu (C.A.)

**Keywords:** aortic stenosis, discordant high gradient aortic stenosis, aortic valve replacement, aortic valve calcification

## Abstract

Aortic stenosis (AS), the most common valvular heart disease, is traditionally graded based on several echocardiographic quantitative parameters, such as aortic valve area (AVA), mean pressure gradient (MPG), and peak jet velocity (Vmax). This systematic review evaluates the clinical significance and prognostic implications of discordant high-gradient AS (DHG-AS), a distinct hemodynamic phenotype characterized by elevated MPG despite a preserved AVA (>1.0 cm^2^). Although often overlooked, DHG-AS presents unique diagnostic and therapeutic challenges, as high gradients remain a strong predictor of adverse outcomes despite moderately reduced AVA. Sixty-three studies were included following rigorous selection and quality assessment of the key studies. Prognostic outcomes across five key studies were discrepant: some showed better survival in DHG-AS compared to concordant high-gradient AS (CHG-AS), while others reported similar or worse outcomes. For instance, a retrospective observational study including 3209 patients with AS found higher mortality in CHG-AS (unadjusted HR: 1.4; 95% CI: 1.1 to 1.7), whereas another retrospective multicenter study including 2724 patients with AS observed worse outcomes in DHG-AS (adjusted HR: 1.59; 95% CI: 1.04 to 2.56). These discrepancies may stem from delays in intervention or heterogeneity in study populations. Despite the diagnostic ambiguity, the presence of high gradients warrants careful evaluation, aggressive risk stratification, and timely management. Current guidelines recommend a multimodal approach combining echocardiography, computed tomography (CT) calcium scoring, transesophageal echocardiography (TEE) planimetry, and, when needed, catheterization. Anatomic AVA assessment by TEE, CT, and cardiac magnetic resonance imaging (CMR) can improve diagnostic accuracy by directly visualizing valve morphology and planimetry-based AVA, helping to clarify the true severity in discordant cases. However, these modalities are limited by factors such as image quality (especially with TEE), radiation exposure and contrast use (in CT), and availability or contraindications (in CMR). Management remains largely based on CHG-AS protocols, with intervention primarily guided by transvalvular gradient and symptom burden. The variability among the different guidelines in defining severity and therapeutic thresholds highlights the need for tailored approaches in DHG-AS. DHG-AS is clinically relevant and associated with substantial prognostic uncertainty. Timely recognition and individualized treatment could improve outcomes in this complex subgroup.

## 1. Introduction

Aortic stenosis (AS) is a progressive valvular disease characterized by the narrowing of the aortic valve, resulting in impaired left ventricular outflow when severe. Severe AS is defined by an aortic valve area (AVA) ≤ 1.0 cm^2^ and/or a mean transaortic pressure gradient (MPG) ≥ 40 mm Hg and/or a peak aortic jet velocity (Vmax) ≥ 4 m/s in normal flow states [1,2,3,4,5]. When all parameters (AVA, MPG, and Vmax) align with each other in the assessment of the degree of stenosis, it is termed concordant AS and is graded depending on AVA, MPG, and Vmax as follows:

- Mild (AVA > 1.5 cm^2^, MPG < 25 mm Hg, or Vmax < 3.0 m/s);
- Moderate (AVA 1.0 to 1.5 cm^2^, MPG 25 to 40 mm Hg, or Vmax 3.0 to 4.0 m/s);
- Severe (AVA < 1.0 cm^2^, MPG > 40 mm Hg, or Vmax > 4.0 m/s) [6].

On the other hand, discordant AS arises when some parameters suggest moderate AS while others suggest a more severe disease. Discordant grading is defined by either a low gradient with an AVA of <1 cm^2^ and an MPG of ≤40 mmHg/Vmax of <4 m/s, or by a high gradient with an AVA of ≥1 cm^2^ and an MPG of ≥40 mmHg/Vmax of ≥4 m/s [7]. Due to this discrepancy between AVA and MPG, discordant AS presents a diagnostic and management challenge, complicating care for these patients [8]. While discordant low-gradient AS (DLG-AS) has been widely studied, there is scarce and controversial literature on the management and outcomes of patients with discordant high-gradient aortic stenosis (DHG-AS) [2,9,10].

It is crucial to distinguish between DHG-AS and concordant high-gradient AS (CHG-AS), as this seldom-discussed AS phenotype has potentially severe adverse outcomes, which could be mitigated by early appropriate intervention [5]. In some studies, patients with DHG-AS appear to have a higher mortality rate compared to those with CHG-AS due to delayed interventions, namely aortic valve replacement (AVR) [9]. AVR may be carried out either surgically or transcatheterally. The hypothesis that an AVA of ≥1.0 cm^2^ under high flow conditions could actually be <1.0 cm^2^ at standard flows, thereby indicating severe concordant AS, has been discussed [2]. Early detection and accurate differentiation are key for improving outcomes and preventing irreversible damage [11]. Mortality in patients with AS can result from the unpredictable progression of the condition, leading to misclassification as non-severe AS and, therefore, delayed treatment, ultimately contributing to worse clinical outcomes [5]. Additionally, comorbid conditions such as previous myocardial infarction and renal disease are linked to increased mortality rates in patients with AS [11]. This review summarizes the current literature on the prevalence, outcomes, and management of DHG-AS, highlighting its clinical significance and the need for specific treatment guidelines for this AS phenotype.

## 2. Methods

### Literature Search Strategy and Inclusion Criteria

To identify relevant studies, we searched PubMed/Medline from January 2010 to December 2024 using the following terms: “aortic valve stenosis” [MeSH Terms] OR “aortic” AND “valve” AND “stenosis” OR “aortic valve stenosis” OR “aortic” AND “stenosis” OR “aortic stenosis” AND “high gradient”.

We included all current relevant original studies that were written in English and involved human subjects. Studies were selected based on their alignment with our review’s objectives, focusing on the epidemiology, pathophysiology, and clinical outcomes of high-gradient AS. Filters were applied to exclude studies involving animal models. Abstracts and full-text articles were reviewed for inclusion by the first two authors (NNB and MTA). Any discrepancies among them were solved by consensus with the rest of the coauthors. References for the included studies were also screened to identify additional relevant articles. The quality of the included studies was evaluated using the Newcastle–Ottawa Quality Assessment Scale for non-randomized studies [12].

## 3. Results

### 3.1. Search Results and Quality Assessment of Included Articles

**Figure 1:**
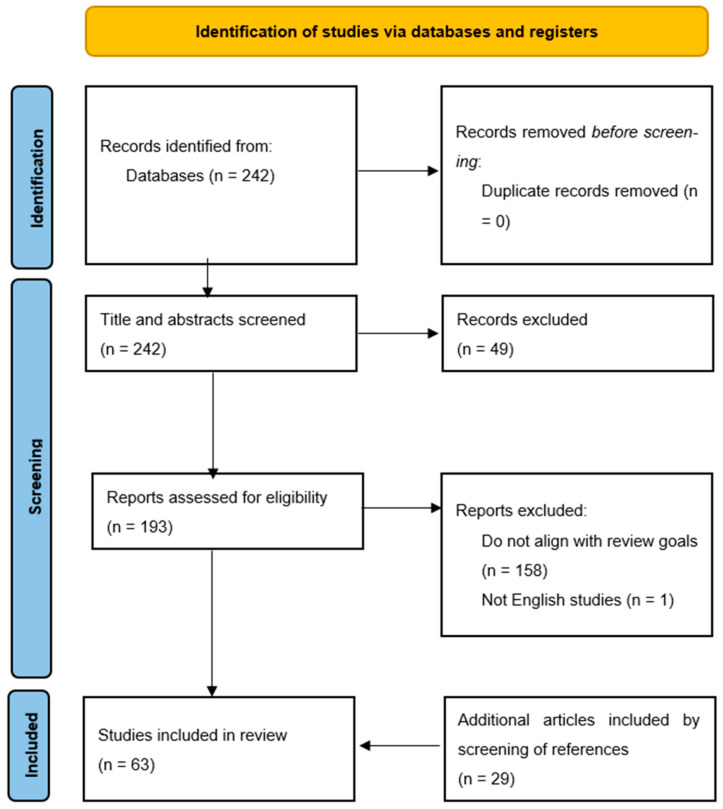
PRISMA 2020 flow diagram showing search strategy for the systematic review [13].

In total, 242 articles were identified. After applying inclusion and exclusion criteria and following the screening of the references of identified manuscripts, 63 articles were included in this systematic review (Figure 1). All studies have consistent ratings for the selection criteria, indicating a robust selection process across the board. Each study has been rated with two stars for comparability, suggesting that the cohorts were well-matched based on design or analysis. The assessment of outcomes shows that most studies adequately followed up with cohorts and assessed outcomes effectively. However, the follow-up adequacy for some studies has not been reported. Overall, all the studies were classified as having a low risk of bias in the criteria mentioned. The results of this quality assessment can be found in Table 1.

### 3.2. Prevalence of DHG-AS

In a single-center retrospective study of 3547 patients, DHG-AS was observed in 163 patients. This represented 4.6% of the entire study population and 11.6% of the patients with high-gradient AS [2]. Other studies have reported lower prevalence rates of 2.1% and 3.5% with DHG-AS, respectively [9,14]. In a separate single-center retrospective study by Ito et al., which included 3209 patients, DHG-AS accounted for 7% of the entire cohort and 14% of patients with high-gradient AS [14]. Another retrospective study conducted on an Asian cohort found discordant grading in nearly 30% of patients with high-gradient AS [8]. Despite the variations in reported prevalence, these findings highlight that DHG-AS is not uncommon and underscore the need for a deeper understanding of this clinical entity.

### 3.3. Pathophysiology and Diagnostic Criteria

#### 3.3.1. Pathophysiology

Despite the standardized definition of severe AS, diagnosing it in clinical practice remains challenging due to frequent discrepancies in measured parameters (30% to 40%) [14]. Given the perioperative risks of surgical and transcatheter AVR, accurate diagnosis is essential. Additionally, timely intervention is crucial to avoid poor outcomes with prolonged active surveillance, which is often the case in patients with DHG-AS [15].

**Figure 2:**
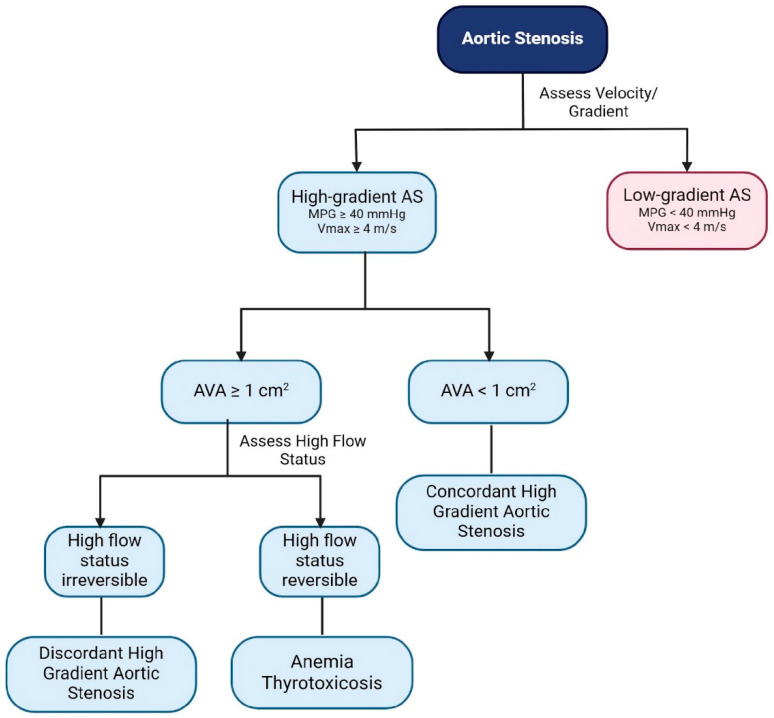
TTE assessment for high-gradient aortic stenosis. TTE: transthoracic echocardiogram; AS: aortic stenosis; MPG: mean transaortic pressure gradient; Vmax: peak aortic jet velocity; AVA: aortic valve area.

Current guidelines emphasize the importance of using a high MPG of >40 mmHg or Vmax of >4 m/s for the diagnosis of severe AS, regardless of the AVA [16,17]. In most circumstances, these thresholds correspond to an AVA of ≤1.0 cm^2^ [18]. High-gradient AS can be subclassified into CHG-AS and DHG-AS (Figure 2), based on AVA reversible conditions associated with high-flow states, such as anemia and thyrotoxicosis, and arteriovenous shunts (Table 2) [14,17].

Patients with DHG-AS often exhibit high stroke volume, which may explain the discordant findings [3]. This phenotype is more common in men, who tend to have larger body surface areas and left ventricular outflow tract (LVOT) diameters [14]. Indexing AVA to body surface area (BSA) using a cut-off of 0.6 cm^2^/m^2^ is recommended for patients with atypically large or small body sizes, particularly those with severe AS. However, this approach is controversial as BSA does not accurately reflect normal AVA in obese patients, and the valve area does not scale with excess body weight. Indexing is important for children, adolescents, and small adults, as it helps distinguish between moderate and severe stenosis [3]. In patients with discordant grading, it can help reconcile discrepancies between valve area and gradient-based severity assessments [25]. Although indexed AVA might suggest similarities between DHG-AS and CHG-AS, DHG-AS may have a more favorable natural history than other AS subtypes [14].

The presence of high flow due to concomitant hemodynamically significant aortic insufficiency is also a contributing factor and can lead to a higher calculated gradient [3]. In addition, Vulesevic et al. suggested that, because the LVOT is an elliptical structure, measurements in such patients may be more aligned with the major axis, leading to a larger measured diameter, which highlights the need for accurate measurement of the LVOT diameter and time-velocity integral (TVI), as the calculated AVA is very sensitive to changes in LVOT diameter measurements [10,26].

#### 3.3.2. Signs and Symptoms

The typical physical sign of severe AS is a harsh, late-peaking systolic murmur, heard loudest over the second right intercostal space and radiating to the carotid arteries [27]. This may be associated with a slow and delayed carotid upstroke, a sustained point of maximal impulse, and a diminished or absent aortic second sound. Symptoms and signs are generally similar between concordant and discordant AS [2]. Around 45% of DHG-AS patients may remain asymptomatic [14]. Symptomatic patients mainly present with shortness of breath, dyspnea, exercise intolerance, angina, and syncope [27]. Further imaging and clinical reasoning help differentiate between them.

#### 3.3.3. Diagnostic Tools and Techniques

**Figure 3:**
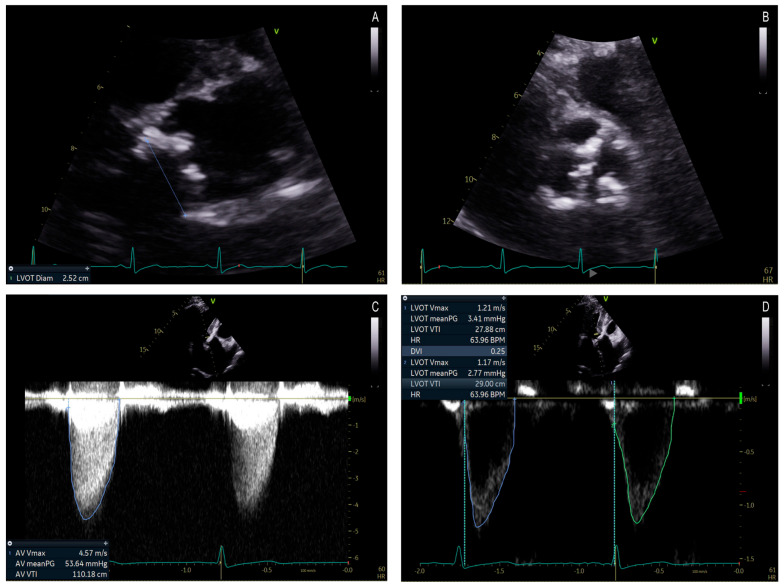
(**A**) 73 year old male patient with discordant high gradient aortic stenosis. Left ventricle outflow tract diameter = 2.52 cm (**A**). (**B**) shows a parasternal short axis view of the aortic valve with severe calcification. Aortic Valve VTI is 110.18 cm, the mean gradient is 53.64 mmHg (**C**), and LVOT VTI is 27.88 cm (**D**). As a result, the aortic valve area by VTI is 1.26 cm^2^, cardiac output is 8.06 L/min, and cardiac index is 4.41 L/min/m^2^.

**Figure 4:**
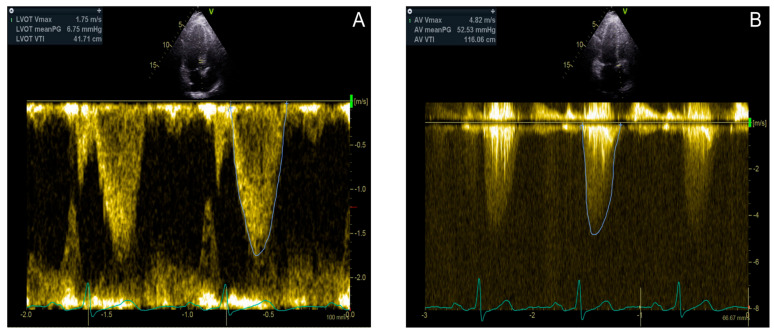
An 80 year old female patient with discordant high gradient aortic stenosis. Left ventricle outflow tract diameter VTI is 41.71 cm (**A**); aortic valve VTI is 116.06 cm, and mean gradient is 52.53 mmHg (**B**). The LVOT diameter is 2.21 cm. According to these measurements, cardiac output is 9.97 L/min, cardiac index is 5.63 L/min/m^2,^ and the aortic valve area by VTI is 1.38 cm^2^.

In patients with suspected AS, a transthoracic echocardiogram (TTE) is indicated and is the first-line diagnostic modality. Doppler echocardiography is used to measure the MPG, Vmax, and AVA (Figure 3 and Figure 4) [3,4,17]. Underestimation of the severity of AS, defined by the transaortic gradient, may occur when Doppler recordings are misaligned and not parallel to the intercept angle of the axis of flow or if the image quality is poor [16]. Conversely, certain conditions can lead to an overestimation of the aortic valve gradient and should, therefore, be considered before diagnosing DHG-AS. These include misidentifying a mitral regurgitation jet, patients with obstructive hypertrophic cardiomyopathy, measurements taken from a post-extrasystolic beat, and the possibility of significant pressure recovery [28].

The pressure recovery phenomenon occurs due to an increase in the kinetic energy of blood flow when it passes through a stenotic aortic valve, which is then converted back into potential energy downstream of the valve [29]. This conversion leads to a partial recovery of pressure in the blood flow after it has passed through the narrowest point of the valve. Significant discrepancies have been observed in gradients measured by Doppler and cardiac catheterization, as this phenomenon can sometimes lead to an overestimation of the severity of valve stenosis when using Doppler ultrasound [30,31]. This is because the pressure recovery correction does not fully account for the differences in measurements [28].

The AVA may be overestimated due to improper spectral Doppler sample volume placement within the zone of flow acceleration when obtaining the LVOT TVI, leading to an overestimation of stroke volume, the numerator of the continuity equation [3]. It is important, in the absence of >moderate mitral regurgitation, to cross-reference the stroke volume measurement(s) made by both Doppler and via either 3D or biplane left ventricular volumes.

**Figure 5:**
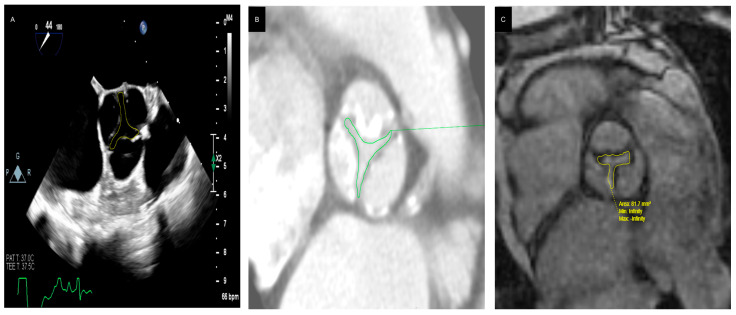
Representative examples illustrating the anatomic area obtained by planimetry in different patients with severe aortic stenosis: TEE (0.8 cm^2^, (**A**)), CT (0.6 cm^2^, (**B**)), and MRI (0.8 cm^2^, (**C**)).

Accurate measurement of the AVA using planimetry can be a useful tool in patients with DHG-AS (Table 3). Although planimetry is less effective with 2D TTE due to extensive valve calcification and imaging plane that may not be aligned with level of the smallest valve orifice, 3D TTE, transesophageal echocardiography (TEE), or multislice computed tomography (MSCT) 3D planimetry can provide more accurate anatomic measurements of the AVA (Figure 5) [26,32,33,34]. Multiple studies have validated the clinical and prognostic relevance of anatomic planimetric aortic valve area assessment in severe AS using TEE, CT, and cardiovascular magnetic resonance imaging (CMR) (Table 4).

Measurement of the anatomic AVA by direct visualization of the valve orifice using TTE or TEE has been evaluated as an alternative to Doppler estimation of flow velocities [3]. Although TTE is an established method for determining the severity of AS, the resolution of TTE may not permit direct planimetry of a stenotic aortic valve. TEE generally obtains better images of the stenotic orifice and is used clinically in suspected AS when there is doubt about the diagnosis or severity. It provides anatomical AVA by direct planimetry and better assessment of the LVOT area. However, obtaining a true short-axis plane is crucial for accurate measurements, and acoustic shadowing from aortic valve calcification can hinder these measurements [35]. In a prospective study involving 67 patients, the correlation coefficient for valve area, computed by planimetry on TEE and by the continuity equation on TTE, was 0.93, with a small percentage difference between the two methods (11%) [32]. The TEE method correlated more closely with the catheterization-determined valve area (using the Gorlin formula) than the TTE value (r = 0.91 vs. 0.84). Planimetry by TEE has also been shown to correlate well with planimetry by computed tomography (CT) [34]. Although TEE can provide superior imaging of aortic valve morphology and complement TTE evaluation in selected cases, caution should be exerted when relying exclusively on TEE assessment of AS for management decisions [36]. The TEE hemodynamic assessment exhibits high sensitivity but low specificity for severe AS compared with the gold standard of TTE assessment during the awake state.

Cardiac CT offers an accurate planimetric assessment of AVA with high spatial resolution. Additionally, aortic valve calcium scoring (AVCS) by cardiac CT is a quantitative and reliable method for assessing AS severity, independent of flow. CT and AVCS accurately classify patients with concordant grading and help identify true severe AS in discordant cases [7]. In discordant grading cases, after verification of potential error measurements, AVCS could be considered complementary to Doppler-echocardiography to confirm AS severity and guide clinical management [37]. In a large, international, multicenter cohort, sex-specific thresholds (women: 1377 AU and men: 2062 AU) emerged as the most powerful predictor of adverse clinical events, both in the cohort as a whole and in patients with discordant echocardiographic findings [38].

CMR is a valuable noninvasive modality for evaluating AS, particularly in complex or discrepant cases. It provides a comprehensive assessment of valvular and myocardial disease [39]. CMR can quantify AS severity comparably to Doppler echocardiography by measuring transvalvular peak velocity and pressure gradient using the simplified Bernoulli equation. Moreover, hemodynamic assessment using four-dimensional (4D) flow MRI offers detailed insights into blood flow patterns, which may aid in risk stratification and support individualized treatment planning to optimize clinical outcomes. In addition to functional assessment, CMR is uniquely capable of detecting myocardial fibrosis, a key prognostic marker in AS, which contributes to improved risk stratification and may guide the timing of aortic valve intervention [40]. Planimetric measurement of the AVA using CMR is accurate and noninvasive, and it offers superior image quality compared to TEE in certain patients [41]. AVA planimetry is typically performed on mid-systolic images and demonstrates comparable accuracy to both TEE and computed tomography coronary angiography (CTCA). However, extensive valvular calcification may introduce artifacts in CMR imaging, potentially limiting its utility for planimetry in patients with moderate to severe leaflet calcification, where CTCA may provide more reliable measurements [42].

**Table 3:** Clinical utility and prognostic relevance of anatomic planimetric area in severe aortic stenosis.

Imaging Modality	Clinical Utility	Prognostic Relevance	Relevance in Discordant High-Gradient Aortic Stenosis (DHG-AS)	Limitations
Transesophageal Echocardiography (TEE)	High-resolution imaging for direct planimetric aortic valve area (AVA) measurement. Preferred when transthoracic echocardiography (TTE) is inconclusive. Excellent for intraoperative and pre-intervention assessments.	TEE-derived AVA closely correlates with invasive measurements. Can help refine grading when Doppler metrics are discordant.	Confirms severity in DHG-AS when TTE shows high gradient but AVA > 1.0 cm^2^. Helps distinguish pseudo-severe from truly severe AS.	Semi-invasive; requires sedation. Imaging may be suboptimal in heavily calcified valves. Operator-dependent and sensitive to the imaging plane.
Computed Tomography (CT)	Provides accurate 3D planimetric AVA in systole. Especially valuable in calcified valves. Enables aortic valve calcium scoring.	CT aortic valve calcium score is a strong independent predictor of mortality and AS severity. CT AVA helps reclassify ambiguous or borderline cases.	In DHG-AS, it supports the diagnosis of severe AS when the calcium burden is high. May guide decision toward earlier intervention.	Involves radiation and iodinated contrast. Lacks flow dynamics; provides only anatomic severity.
Cardiac Magnetic Resonance (CMR)	Noninvasive AVA planimetry without radiation. Superior for left ventricle function and myocardial fibrosis assessment. Useful in patients with contraindications to CT or poor echo windows.	Detection of myocardial fibrosis strongly correlates with adverse prognosis, independent of AVA. AVA planimetry by CMR may support risk assessment.	In DHG-AS, CMR helps integrate valve severity with myocardial disease burden and may guide the timing of aortic valve intervention. AVA + fibrosis information enhances stratification for AVR.	Limited availability and cost. Lower spatial resolution than CT. Not routinely used for valve area assessment in clinical practice.

**Table 4:** Studies that have validated the clinical and prognostic significance of anatomic planimetric area assessment via TEE, CT, and CMR in severe aortic stenosis.

Study	Type of Study	Imaging Modality	Sample Size	Key Findings	Clinical Implications
Habis et al. [43]	Prospective Observational Study	64-slice CT vs. TTE	52	The aortic orifice area measured by 64-slice CT correlated well with the effective area (r = 0.76; *p* < 0.0001), but was significantly greater, with a systematic overestimation (0.132 cm^2^).	CT planimetry allows accurate classification of AS severity comparable to echocardiographic methods.
John et al. [44]	Prospective Observational Study	MRI vs. TEE vs. Catheterization	40	Mean absolute differences in AVA were 0.02 cm^2^ for MRI versus TEE, 0.27 cm^2^ for MRI versus catheter, and 0.25 cm^2^ for TEE versus catheter. Correlations for AVA_max_ were r = 0.96 between MRI and TEE, r = 0.47 between TEE and catheter, and r = 0.44 between MRI and catheter.	Magnetic resonance planimetry of the AVA correlates well with TEE and less well with the catheter-derived AVA. MRI planimetry of the AVA may provide an accurate and noninvasive alternative to invasive techniques and TTE.
Feuchtner et al. [45]	Prospective Observational Study	64-slice CT vs. TTE and TEE	36	CT AVA planimetry (1.11 ± 0.42 cm^2^) showed a good correlation with TTE (1.05 ± 0.42 cm^2^) (r = 0.88, *p* < 0.001) as well as with TEE (1.41 ± 1.61 cm^2^) (r = 0.99, *p* < 0.0001).	MSCT allows accurate planimetry of the AVA in patients with aortic stenosis comparable to both TTE and TEE.
Westermann et al. [46]	Prospective Observational Study	MSCT and MRI vs. TEE	27	Excellent correlation between MSCT and MRI (r = 0.99). The mean AVAs on both MSCT and MRI were systematically larger than on TTE (0.88 ± 0.28 cm^2^, *p* < 0.001 each)	MSCT and MRI have shown excellent correlation in AVA planimetry and similar accuracy in grading aortic valve stenosis.
Knobelsdorff-Brenkenhoff et al. [47]	Prospective Observational Study	CMR vs. TTE and TEE	65	Correlations of the AVA by CMR with TTE (r = 0.82) and CMR with TEE (r = 0.92) were significant.	CMR provides estimation of AVA with a close correlation to echocardiography and has low observer dependency.
Paelinck et al. [48]	Prospective Observational Study	MRI vs Catheterization vs. TTE and TEE	24	No differences in AVA were found among MRI, Doppler echocardiography, and three-dimensional TTE compared with catheterization (*p* = NS).	MRI planimetry, Doppler, and three-dimensional TTE provide an accurate estimate of the AVA compared to catheterization.
Alkadhi et al. [49]	Prospective Observational Study	16-detector row CT vs. TTE and TEE	40	Significant correlations were present between AVA(CT) and AVA(TEE) (r = 0.99, *p* < 0.001), AVA(CT) and AVA(TTE) (r = 0.95, *p* < 0.001). Mean Differences were −0.08 cm^2^ for AVA(CT) vs. AVA(TEE) and 0.06 cm^2^ for AVA(CT) vs. AVA(TTE).	Planimetric measurements of AVA using a 16-detector row CT allow for the classification of AS, similar to echocardiographic methods.

Abbreviations: TEE: transesophageal echocardiography; CT: computed tomography; CMR: cardiac magnetic resonance imaging; MRI: magnetic resonance imaging; AS: aortic stenosis; AVA: aortic valve area; MSCT: multislice CT.

### 3.4. Prognosis and Outcomes of DHG-AS

AS is the most common valvular disease in developed nations and is associated with significantly reduced long-term survival [50]. While patients with DHG-AS may have a more favorable prognosis compared to CHG-AS in some studies, they still experience higher mortality rates compared to the general population, underscoring the severity of their condition [8,14]. The prognosis of DHG-AS compared to CHG-AS remains inconsistent, with some studies showing similar event-free survival rates and others reporting worse outcomes due to delays in or lack of AVR (Table 5) [2,14].

A study involving 3209 patients, including 230 individuals with DHG-AS (7.2%), found that all-cause mortality over a median follow-up of 944 days was higher in CHG-AS (HR: 1.4; 95% CI: 1.1–1.7) and moderate AS (HR: 1.4; 95% CI: 1.1–1.7) compared to DHG-AS [14]. However, after adjustment for age, comorbidities, bicuspid valve, and cardiac function, these differences were no longer significant.

Among DHG-AS patients, those who underwent AVR had significantly better survival outcomes than those who did not (*p* < 0.001). This suggests that despite a relatively better prognosis, AVR may remain beneficial, improving survival and reducing all-cause and cardiovascular mortality [14]. In addition, DHG-AS patients exhibited lower heart failure prevalence and better-preserved cardiac function, contributing to improved outcomes. The findings highlight the importance of recognizing the severity of AS in patients with an AVA > 1.0 cm^2^ and the potential life-saving benefits of AVR.

#### Comparative Studies on DHG-AS and CHG-AS

An Asian cohort study of 467 patients from 2010 to 2015 found that DHG-AS patients had a more favorable prognosis than CHG-AS patients, despite both groups having higher AVR rates than moderate AS patients [8]. CHG-AS was significantly associated with increased all-cause mortality and congestive heart failure (CHF) admissions compared to moderate AS, while DHG-AS was not. This study concluded that MPG was a crucial predictor of outcomes, and after excluding reversible high-flow states, DHG-AS patients had better prognostic outcomes than CHG-AS patients.

A retrospective observational study involving 3547 adults from 2005 to 2015 found DHG-AS to be relatively common (>10%) and was associated with a poor prognosis. Mortality rates were similar between DHG-AS and CHG-AS patients, regardless of AVA. However, survival without AVR was slightly better in DHG-AS. The poor outcomes in DHG-AS were attributed to high-pressure gradients increasing afterload and valvular damage, irrespective of the AVA [51,52].

DHG-AS patients exhibited high AVCS, comparable to CHG-AS patients, which may contribute to disease progression and higher mortality risk [2,5,53,54]. AVCS may help re-stratify grading of AS severity when TTE measurements show discordance [55].

The only prospective study to date focusing on DHG-AS outcomes included 234 patients (155 with moderate AS, 56 with CHG-AS, and 23 patients with DHG-AS) [10]. The study found that DHG-AS patients had event-free survival rates comparable to those of CHG-AS, despite having larger AVAs. Both groups had similar AVCS, supporting the notion that MPG, rather than AVA, is a key prognostic indicator. The study reinforced the importance of using transvalvular gradients rather than AVA in assessing AS severity and guiding clinical decision-making in patients with high-gradient AS.

Among 2724 patients (2606 with CHG-AS, 118 with DHG-AS) evaluated between 2000 and 2018, those with DHG-AS were typically younger, predominantly male, and had fewer comorbidities but exhibited larger LV dimensions [9]. Overall, DHG-AS patients had higher mortality rates, likely due to either delayed or less frequent AVR. Even after propensity matching, the 5-year survival rate for DHG-AS patients remained lower, reinforcing the need for timely AVR to improve outcomes [9]. Large prospective interventional studies need to be conducted to better understand the prognostic differences between DHG-AS and CHG-AS. Overall, the current literature presents conflicting findings regarding the prognosis of DHG-AS, with its long-term risks comparable to severe CHG-AS; thus, these patients should be carefully monitored, and early management should be considered.

### 3.5. Management of DHG-AS

Current guidelines recommend AVR for patients with a Vmax of ≥4 m/s or an MPG of ≥40 mmHg regardless of AVA, if they are symptomatic or have an LVEF of <50% (Table 6) [56]. However, specific recommendations for managing DHG-AS are lacking due to limited data, and further research is necessary to evaluate the best approach to therapy in DHS-AS patients.

The management of DHG-AS begins with confirming that the elevated gradient is not caused by high-flow states. Additionally, reversible sources of discordance—such as echocardiographic measurement inconsistencies and variations in body size—should be addressed [8]. In individuals with a bicuspid aortic valve (BAV), the LVOT diameter is often, but not always, larger (>2.4 cm) compared to those with a tricuspid aortic valve [57]. Thus, discordant grading is often observed. These patients are typically classified as having severe AS and tend to undergo interventions earlier [7]. Therefore, AVR is more frequently performed in patients with bicuspid AS compared to patients with tricuspid AS [81.3% (n = 74) vs. 58.3% (n = 81)] [14]. Once these factors are ruled out, accurate anatomic assessment of valve area using TTE, TEE 3D planimetry, or MSCT can help clarify AS severity before considering AVR [32,34]. The choice between surgical AVR and transcatheter AVR for patients with DHG-AS is similar to that for patients with CHG-AS, depending on the individual patient’s characteristics, risks, and preferences [58]. A thorough evaluation by a multidisciplinary team is essential to determine the best approach on a case-by-case basis, with SAVR being the first line of treatment, and patients with higher surgical risk being considered for a transcatheter approach [59]. The decision-making process regarding the superiority of one intervention over the other for patients with DHG-AS remains an uncharted territory that requires further exploration.

In a retrospective study of 3209 patients, various AS hemodynamic profiles revealed that DHG-AS patients who underwent AVR had a lower cumulative incidence of all-cause mortality and cardiac mortality compared to patients who did not (Gray’s Test: *p* = 0.001 and *p* = 0.001, respectively) [14]. Despite the reported benefits, AVR is often delayed in DHG-AS patients, leading to worse outcomes. A propensity-matched study of 90 DHG-AS patients found a higher 5-year cumulative mortality rate compared to CHG-AS patients (22% vs. 9%, *p* = 0.030). This mortality difference was mainly driven by lower AVR rates among DHG-AS patients compared to CHG-AS patients, indicating that DHG-AS is often managed conservatively, with intervention postponed due to diagnostic and decision-making challenges [9].

The prognosis of DHG-AS remains debated. Some studies suggest that DHG-AS patients have a more favorable prognosis than CHG-AS patients, while others report similar outcomes between the two groups [8,9]. Studies with longer follow-up periods suggest that the initial survival advantages observed in DHG-AS patients may diminish over time without intervention [2,9].

Given these conflicting findings, clinical guidelines should provide specific recommendations for DHG-AS rather than grouping these patients within the general high-gradient AS management protocols. Further prospective studies are essential to refine treatment strategies and determine the optimal timing of AVR for this patient population.

**Table 6:** Summary of diagnostic and management guidelines for DHG-AS.

	American Guidelines ^a^	European Guidelines ^b^	Canadian Guidelines ^c^	Japanese Guidelines ^d^
Diagnosis	TTE is the initial diagnostic tool. When AVA and hemodynamic parameters are discordant, the LVOT/AV peak velocity ratio is useful, as it reduces errors caused by variability in LVOT size and body size. Cardiac CT or TEE planimetry is recommended to confirm AS severity. If noninvasive tests are inconclusive, cardiac catheterization is advised for definitive evaluation.	TTE is the standard initial diagnostic tool. In cases of DHG-AS, additional parameters and factors should be considered, such as LVOT/AV velocity ratio, degree of valve calcification, and high-flow conditions. For asymptomatic patients, exercise testing is recommended. Cardiac CT (with calcium scoring) aids in AVA assessment and prognosis. CT or TEE planimetry confirms AS severity. CMR may be considered for detecting myocardial fibrosis, a key determinant of poor prognosis in AS patients. Cardiac catheterization is a last resort when noninvasive tests are inconclusive.	A multimodality imaging approach similar to the American and European guidelines is recommended to better assess the severity of AS.	Japanese guidelines classify AS severity mainly by AVA (severe if <1.0 cm^2^), unlike others that prioritize pressure gradients. TTE is the initial tool; severe AS is divided into high- and low-gradient types. If the TTE is inconclusive, a TEE is recommended for a detailed assessment. CMR may be utilized to provide prognostic information, particularly regarding myocardial fibrosis. Cardiac catheterization is used preoperatively when noninvasive tests are inconclusive or hemodynamic confirmation is needed.
Management	Guidelines lack specific DHG-AS recommendations, so CHG-AS criteria are used. Severity is primarily based on the mean gradient and peak velocity, rather than AVA. AVR is recommended for severe AS (mean gradient of ≥40 mmHg or peak velocity of ≥4.0 m/s) in the presence of symptoms, a drop in blood pressure during exercise testing, peak velocity of ≥5.0 m/s, reduced LVEF, or elevated BNPs. In asymptomatic patients, AVR is also recommended in those undergoing other cardiac surgeries, and in cases where there is rapid disease progression (increase in peak velocity of >0.3 m/s/year). Both U.S. and European guidelines recommend stratifying patients into TAVR, SAVR, or intermediate groups, with decisions for the latter based on individualized assessment and shared decision-making; however, their stratification approaches differ.		The Canadian guidelines adopt a treatment approach that closely aligns with the European recommendations, with the notable distinction of placing greater emphasis on a comprehensive, individualized assessment of multiple factors (age, STS score, etc.) by a multidisciplinary heart team to guide the choice between SAVR and TAVR.	The Japanese guidelines support aortic valve intervention based on clinical indications that are broadly aligned with American and European recommendations. However, differences exist in the criteria used to define disease severity, particularly the emphasis on AVA rather than solely pressure gradients. Intervention is indicated for symptomatic severe AS or asymptomatic patients with any of the following: LVEF of <50%, exercise-induced symptoms or blood pressure drop, peak velocity of ≥5.0 m/s, rapid disease progression, mean gradient of ≥60 mmHg, sPAP of ≥60 mmHg, or elevated BNP. The choice between SAVR and TAVR is made by a multidisciplinary heart team, considering multiple factors (age, comorbidities, JapanSCORE, anatomical characteristics, and institutional expertise, etc.).
	SAVR is preferred in patients younger than 65 years with an expected survival of at least 20 years. In contrast, transcatheter aortic valve replacement TAVR is favored in patients older than 80 years with a life expectancy of less than 10 years.	The European guidelines apply a higher age threshold than the American ones for surgical aortic valve replacement SAVR recommending it for patients younger than 75 years with an STS-PROM/EuroSCORE below 4. Conversely, TAVR is preferred in patients older than 75 years with an STS-PROM/EuroSCORE greater than 8.		

AS: aortic stenosis; AV: aortic valve; AVA: aortic valve area; AVR: aortic valve replacement; BNP: brain natriuretic peptide; CHG-AS: concordant high-gradient aortic stenosis; CMR: cardiac magnetic resonance imaging; CT: computed tomography; DHG-AS: discordant high-gradient aortic stenosis; LVEF: left ventricular ejection fraction; LVOT: left ventricular outflow tract; NTproBNP: N-terminal pro-B-type natriuretic peptide; SAVR: surgical aortic valve replacement; TAVR: transcatheter aortic valve replacement; sPAP: systolic pulmonary arterial hypertension; TEE: transesophageal echocardiography; TTE: transthoracic echocardiography. ^a^ [16], ^b^ [17], ^c^ [60], ^d^ [61]

## 4. Conclusions

DHG-AS is a clinically relevant but often under-recognized hemodynamic subtype of aortic stenosis, marked by elevated gradients despite a preserved AVA. Current evidence presents conflicting data on its prognosis, with some studies reporting more favorable outcomes than CHG-AS, while others indicate similar or worse outcomes. The high aortic valve calcium score (AVCS) observed in DHG-AS supports the need for close monitoring and timely intervention. Accurate assessment is essential given the mismatch between anatomic and hemodynamic severity. Planimetric imaging using TEE, CCT, and CMR provides valuable diagnostic insights and supports more informed decision-making, although each modality has its limitations. Variability in guideline definitions and treatment thresholds further highlights the need for individualized evaluation and management. Large-scale prospective studies are needed to clarify prognosis and optimize treatment strategies for this complex patient subgroup.

## Figures and Tables

**Table 1:** Quality assessment of included studies using the Newcastle–Ottawa Quality Assessment Scale in non-randomized studies.

Study	Selection				Comparability	Outcome		
	Representativeness of the Exposed Cohort	Selection of the Non-Exposed Cohort	Ascertainment of Exposure	Demonstration of the Outcome of Interest Was Not Present at the Start of the Study	Comparability of Cohorts on the Basis of the Design or Analysis	Assessment of Outcome	Was Follow-Up Long Enough for Outcomes to Occur?	Adequacy of Follow-Up of Cohorts
Ito, S. et al. (2024) [14]	*	*	*	*	**	*	*	*
Unger, P. et al. (2024) [2]	*	*	*	*	**	*	*	*
Chew, N. W. S. et al. (2022) [8]	*	*	*	*	**	*	*	*
Bohbot, Y. et al. (2021) [9]	*	*	*	*	**	Not reported	*	*
Vulesevic, B. et al. (2020) [10]	*	*	*	*	**	Not reported	*	*

* indicated that criteria has been met, according to the Newcastle–Ottawa Quality Assessment Scale. For the criteria of “Comparability”, ** indicated that criteria has been met. If criteria was not reported in study, “Not reported” is noted in table.

**Table 2:** Summary of conditions that result in high flow status.

Causes of High Flow Status	The Threshold at Which There Is a Significant Increase in Cardiac Index (>4 L/min/m^2^)
Anemia	Severe anemia (Hb < 7 g/dL ^a^)
Renal failure	Stage 4–5 chronic kidney disease (eGFR < 30 mL/min ^b^)
Tachycardia	>110 bpm ^c^
Hyperthyroidism	Moderate hyperthyroidism: TSH < 0.01 mIU/L Free T4 > 2.5 ng/dL Free T3 > 8 pg/mL ^d^
Liver failure	Stage 4 liver disease (Child-Pugh class C): ^e,f^ INR > 2.3 Albumin < 2.8 g/dL Bilirubin > 3 mg/dL

Abbreviations: ALT: alanine aminotransferase; AST: aspartate aminotransferase; INR: international normalized ratio; GFR: glomerular filtration rate; Hb: hemoglobin; TSH: thyroid-stimulating hormone. ^a^ [19], ^b^ [20], ^c^ [21], ^d^ [22], ^e^ [23], ^f^ [24].

**Table 5:** Summary of available studies that focused on outcomes of patients with DHG-AS.

Study	Type of Study	Population Size (% of Dhg-As Among High-Gradient As)	Outcome
Ito, S. et al. (2024) [14]	Retrospective observational, single-center study	3209 (13.5%)	All-cause mortality was higher in CHG-AS patients compared to patients with DHG-AS (unadjusted HR: 1.4; 95% CI: 1.1 to 1.7)
Unger, P. et al. (2024) [2]	Retrospective observational, single-center study	3547 (11.6%)	The mortality rate for DHG-AS patients was similar to that of those with CHG-AS (adjusted HR: 0.98, 95% CI: 0.66 to 1.44; *p* = 0.91)
Chew, N. W. S. et al. (2022) [8]	Retrospective observational, single-center study	467 (30.8%)	CHG-AS patients were significantly associated with all-cause mortality (adjusted HR: 3.082, 95% CI: 1.479 to 6.420; *p* = 0.003) and CHF admissions (adjusted HR: 12.728, 95% CI: 2.922 to 55.440; *p* = 0.001), but DHG-AS patients were not in reference to moderate AS
Bohbot, Y. et al. (2021) [9]	Retrospective observational, multicenter study	2724 (4.3%)	DHG-AS patients had higher mortality rates compared to CHG-AS (adjusted HR: 1.59, 95% CI:1.04 to 2.56)
Vulesevic, B. et al. (2020) [10]	Prospective observational, single-center study	234 (29.1%)	Event-free (sudden death, congestive heart failure, or new onset of symptoms of dyspnea, angina, or syncope) survival of patients with CHG-AS and DHG-AS was similar (*p* = 0.45)

Abbreviations: CHG-AS: concordant high-gradient aortic stenosis; DHG-AS: discordant high-gradient aortic stenosis; HR: hazard ratio; CI: confidence interval.

## Abbreviations

The following abbreviations are used in this manuscript:

AS	Aortic stenosis
DHG-AS	Discordant high-gradient aortic stenosis
CHG-AS	Concordant high-gradient aortic stenosis
AVA	Aortic valve area
MPG	Mean transaortic pressure gradient
Vmax	Peak aortic jet velocity
